# RESCUE-AR: Responsive Sensor-Fusion for Civil-Protection and Disaster Response Using Mobile Augmented Reality

**DOI:** 10.3390/s26206489

**Published:** 2026-10-13

**Authors:** Rui Miguel Pascoal, José Manuel Naranjo Gómez, Alessandro Pinheiro, Mauro Cesar Cantarino Gil

**Affiliations:** 1 Information Sciences, Technologies and Architecture Research Centre—Software Systems Engineering, ISCTE—University Institute of Lisbon, 1649-026 Lisbon, Portugal; 2 Atlântica University Institute, 2730-036 Barcarena, Portugal; mgil@uatlantica.pt; 3 IADE, European University, 1200-649 Lisbon, Portugal; 4 University of Extremadura, 06006 Badajoz, Spain; 5 Valoriza—Research Centre for Endogenous Resource Valorization, 7300-555 Portalegre, Portugal; 6 ISTEC—Higher Institute of Advanced Technologies of Lisbon, 1900-221 Lisbon, Portugal; 7 Autonomous University of Lisbon, 1169-023 Lisbon, Portugal; 8 C-MAST—Centre for Mechanical and Aerospace Science and Technologies, Branch at Atlântica University Institute, 2730-036 Barcarena, Portugal

**Keywords:** augmented reality, sensor fusion, civil protection, disaster response, decision support, usability evaluation

## Abstract

Rapid situational awareness is a cornerstone of effective civil protection and disaster response, demanding highly resilient localization and decision-support capabilities under challenging environmental conditions. This paper presents RESCUE-AR (Responsive Sensor-Fusion for Civil-Protection and Disaster Response Using Mobile Augmented Reality), a conceptual and methodological framework for mobile Augmented Reality (AR) systems designed to support emergency operations through heterogeneous and adaptive sensor fusion. This work formalizes the mathematical and algorithmic architecture of the RESCUE-AR framework, establishing an adaptive multi-sensor measurement fusion strategy, integrating Visual SLAM, IMU, depth sensing, and GNSS data to enhance robustness in environments affected by occlusion, signal degradation, and dynamic change. The framework is grounded in a systematic literature review that identifies the limitations of single-sensor and fixed-weight fusion approaches in disaster scenarios and motivates the adoption of adaptive fusion strategies. An initial user-centered evaluation is conducted through a structured approach, employing standardized questionnaires to assess usability, cognitive workload, anticipated operational trust, and framework suitability for first responders. The results indicate strong perceived utility, reduced cognitive load, and high confidence in AR-assisted decision-making, supporting the potential of adaptive sensor-fusion-based mobile AR as a viable and scalable decision-support tool for civil protection and disaster response.

## 1. Introduction

Civil protection operations require rapid, accurate situational awareness to minimize response times and mitigate risks in dynamic, often hazardous, environments. Traditional methods of data collection and spatial visualization, typically relying on two-dimensional maps or static Geographic Information Systems (GIS), frequently fail to provide the necessary context and precision required by first responders operating under high-stress conditions [1]. This critical need for a robust, field-deployable decision-support tool has driven significant interest in Mobile Augmented Reality (MAR) solutions, which have shown promising value across various outdoor applications [2] and geospatial measurement frameworks [3].

However, the effective deployment of mobile AR in civil protection faces a critical technological hurdle: achieving and maintaining precise localization and tracking accuracy in environments subject to signal degradation, poor visibility (e.g., smoke, dust), and lack of distinct visual features. This localization challenge is central to methods for both mobile devices and autonomous vehicles [4]. While the evolution of Simultaneous Localization and Mapping (SLAM) is foundational, systems relying on a single sensor or fixed-weight multi-sensor fusion often prove unreliable when sensor confidence fluctuates rapidly [5], particularly in dynamic environments prone to occlusion and feature loss [6].

While recent research demonstrates that adaptive fusion strategies leveraging Machine Learning (e.g., LSTM networks) can dynamically adjust sensor contributions [7], these models often impose heavy computational and thermal burdens, rendering them unsuitable for continuous execution on edge mobile devices in high-stress disaster zones. To address this, the methodological novelty of RESCUE-AR lies in proposing a computationally lightweight, deterministic covariance scaling architecture. Unlike standard fixed-weight Kalman filtering, our approach introduces context-aware analytical functions that dynamically scale sensor covariance matrices based on instantaneous environmental stressors, bridging the gap between rigid fixed-weight models and resource-heavy ML approaches. This work introduces RESCUE-AR (Responsive Sensor-Fusion for Civil-Protection and Disaster Response Using Mobile Augmented Reality), a mobile AR framework specifically engineered to address this gap. RESCUE-AR integrates data from heterogeneous smartphone sensors (Visual, Inertial, and Geospatial) using an adaptive fusion engine is designed to support first responders with highly accurate geospatial estimates and Augmented Reality visualizations. The system’s design emphasizes interoperability with official GIS platforms and usability in high-pressure scenarios [1]. While our previous work, EfMAR [3], established a foundational mobile AR framework for geospatial measurements under nominal outdoor conditions, RESCUE-AR represents a distinct advancement strictly tailored for disaster response. Unlike EfMAR, which relies on standard tracking protocols, RESCUE-AR introduces a novel adaptive multi-sensor fusion architecture explicitly designed to maintain spatial tracking integrity under severe environmental degradation (e.g., GNSS denial, visual occlusion). Furthermore, this study contributes a specialized human-centric evaluation focusing on cognitive workload and operational trust under simulated crisis scenarios.

### Gaps in the Literature

The analysis of the state of the art reveals three primary limitations that the RESCUE-AR framework is designed to address. (i) Reliability of Localization in Harsh Environments: Existing mobile AR solutions rely predominantly on standard Visual–Inertial or fixed-weight multi-sensor fusion methods [5,6]. These methods fail to provide the necessary robust and continuous positional accuracy required by first responders when sensor reliability rapidly degrades due to smoke, dust, or GNSS signal denial. (ii) Lack of Adaptive Geospatial Robustness: While multi-sensor fusion is utilized in other domains [4], the application of adaptive sensor fusion methodologies, which dynamically adjust sensor weights based on real-time uncertainty, has not been systematically formalized or validated within the context of mission-critical civil protection operations [7]. (iii) Integration and Usability in Operational Scenarios: There is a persistent need for AR frameworks that move beyond technical feasibility to ensure seamless, real-time interoperability with official civil protection GIS systems and demonstrate empirically improved usability and faster decision-making under genuine operational stress [1].

To establish a robust foundation for multi-sensor instrumentation in critical environments, this work focuses on the deterministic and stochastic modeling of sensor boundaries prior to hardware deployment, rather than a fixed physical prototype implementation. Instead, the computational contribution of RESCUE-AR is positioned at the formalization of the adaptive covariance scaling laws and structural sensor-fusion layers. This methodology provides the analytical grounding and user-centered validation necessary to support and enhance spatial tracking stability, establishing the theoretical precursors, mathematical bounds, and perceived operational measurement value required before large-scale physical field deployment.

To validate the framework’s effectiveness and contribution, this study seeks to answer the following research questions (RQs), aligned with a framework-driven and user-centered evaluation approach: RQ1: How can a heterogeneous adaptive sensor fusion architecture theoretically enhance the robustness and reliability of mobile AR localization in dynamic and challenging environments typical of disaster response, when compared to single-sensor and fixed-weight fusion approaches identified in the literature? RQ2: To what extent does the RESCUE-AR framework support decision-making, situational awareness, and coordination for first responders, as perceived through usability, cognitive workload, trust, and clarity of AR-based information? RQ3: How can the modular architecture of RESCUE-AR ensure seamless interoperability and real-time data exchange with existing GIS and cloud-based civil protection management systems?

The remainder of this work is structured as follows: Section 2 reviews related work, focusing on the state of the art in mobile Augmented Reality and multi-sensor fusion for civil protection and disaster response. Section 3 details the modular architecture of the RESCUE-AR framework. Section 4 presents the methodology, including a systematic literature review, a framework-driven evaluation design, and user-centered validation through standardized questionnaires. Section 5 discusses the findings derived from the literature synthesis and user evaluation, emphasizing perceived utility, usability, trust, and operational relevance. Finally, Section 6 concludes the paper and outlines directions for future research.

## 2. Related Work

### 2.1. Mobile AR for Disaster Response and Usability Challenges

Interactive and wearable AR applications have expanded significantly across educational and public safety domains [8,9]. Activity recognition and user interaction models in outdoor environments further emphasize the need for responsive interfaces [10]. Moreover, edge-computing and cloudlet architectures [11,12] provide crucial support for mobile AR bandwidth demands. Comprehensive surveys on AR foundational concepts, user interfaces, and mobile paradigms [13,14,15,16,17] consistently highlight that technical accuracy must be tightly coupled with usability and reduced cognitive load to serve a mission-critical purpose.

While the foundational concepts of Augmented Reality have been established for decades, the transition to mobile, field-deployed AR for emergency management introduces unique challenges related to the operational environment and user experience. Systems designed for civil protection typically aim to improve Scene Perception by overlaying crucial, context-relevant information, such as danger perimeters, pathfinding, and the position of critical assets, onto the responder’s view. Recent literature has focused specifically on operationalizing AR for public safety, acknowledging that the technical system must function robustly under high-stress conditions.

The general utility and perceived quality of experience (QoE) of mobile AR in diverse outdoor applications have been thoroughly explored [2]. Furthermore, studies explicitly focused on the application domain, such as the work by Gomes et al. [1], detail the development of mobile AR georeferenced systems tailored for emergency response operations. Complementarily, recent frameworks like EfMAR [3] demonstrate the feasibility and precision of outdoor mobile AR for accurate geospatial measurements in unconstrained environments. These highlight the essential need to optimize data visualization and enhance user performance under disaster conditions. Crucially, the practical validation of such systems reveals that technical accuracy must be tightly coupled with usability and reduced cognitive load to truly serve a mission-critical purpose. The implementation of touch-less interactive AR, for instance, underscores the continuous effort to improve user interaction and reduce distractions in field operations [18]. This context directly validates the design requirements of the RESCUE-AR framework.

Furthermore, the theoretical framework for situational awareness in field operations is increasingly supported by next-generation virtual exposure and immersive spatial technologies. Moving beyond static 2D displays, contemporary immersive paradigms construct realistic, spatially anchored 3D situational models that enhance scene perception and operational risk assessment. Integrating these spatial exposure principles into mobile AR enables RESCUE-AR to project dynamic hazard perimeters and evacuation routes with high contextual fidelity, directly supporting first responders’ spatial cognition under high-stress scenarios.

### 2.2. Limitations of Single-Sensor SLAM and the Need for Multi-Sensor Fusion

Visual SLAM survey literature [19] and multi-sensor fusion reviews [18] confirm that single-sensor approaches suffer from rapid failure under occlusion or feature scarcity. Marker-based fiducial techniques [20] or specialized industrial optical scanners [21] offer localized precision but lack scalability in unconstrained disaster zones.

The fundamental technical hurdle for effective outdoor mobile AR is maintaining precise and robust device localization. Simultaneous Localization and Mapping (SLAM) is the cornerstone of modern AR tracking, yet its reliability is contingent upon the quality of sensor data. The challenge of localization is widely recognized across domains, including in the comprehensive surveys for autonomous vehicles [4]. SLAM methods relying on a single sensor (e.g., pure Visual SLAM or pure GNSS) perform sub-optimally or fail outright in environments typical of a disaster area, scenarios characterized by heavy feature scarcity, smoke/dust occlusion, or complete GNSS (Global Navigation Satellite System) signal denial.

Specifically, maintaining feature tracking under dynamic conditions and environmental occlusion remains a major challenge in SLAM [6]. To mitigate these difficulties, the current state of the art overwhelmingly points to multi-sensor fusion as the only viable strategy. This approach strategically combines data from heterogeneous sensors (Visual Odometry, LiDAR/Depth Sensing, IMU, and GNSS) to compensate for the critical failure modes of any single sensor.

The necessity for this shift is comprehensively reviewed by Wang et al. [5], who track the evolution of SLAM and emphasize that heterogeneous fusion is the key to achieving the required resilience for complex and dynamic outdoor scenarios. Their findings reinforce the critical gap that single-sensor AR frameworks present in civil protection, providing the foundational technical justification for the multi-sensor core of the RESCUE-AR architecture.

### 2.3. Justifying Adaptive Fusion: The Gap in Fixed-Weight Methods

While multi-sensor fusion is acknowledged as necessary [5], the majority of deployed mobile AR systems still rely on fixed-weight fusion algorithms (e.g., standard Kalman Filters). These methods, by nature, assume a stable and predictable sensor noise profile. However, in emergency scenarios, sensor accuracy is volatile: the visual system’s confidence plummets in low light, while GNSS signals can be instantly jammed or lost near damaged structures. Fixed-weight systems cannot dynamically adapt to these real-time shifts in sensor reliability, often leading to rapid drift and compromised AR visualization.

The most recent research frontier demonstrates that the next step in robust localization is adaptive sensor fusion, which dynamically adjusts the contribution (or weight) of each sensor based on real-time confidence or environmental context.

This principle is strongly supported by recent advancements, such as the work by Irfan et al. [7], which successfully implemented a Self-Adaptive Multi-Sensor Fusion model powered by Deep Learning (specifically, an LSTM network). Their results showcase significantly enhanced robustness in challenging environments by adaptively balancing sensor contributions. This directly addresses the final, critical gap: RESCUE-AR moves beyond the limitations of fixed-weight multi-sensor fusion by implementing a comparable adaptive strategy, ensuring the framework maintains maximum positioning accuracy and mission reliability.

Furthermore, to prevent cognitive saturation during high-stress operations, adaptive user interface filtering and machine-learning-driven information overload reduction mechanisms [22,23] are essential. Complementarily, recent progress in view synthesis, monocular metric depth estimation, and mobile ToF/LiDAR sensor fusion [24,25,26] provides the algorithmic foundation for the depth-aware spatial overlays integrated into the RESCUE-AR framework.

## 3. RESCUE-AR Framework

**Figure 1:**
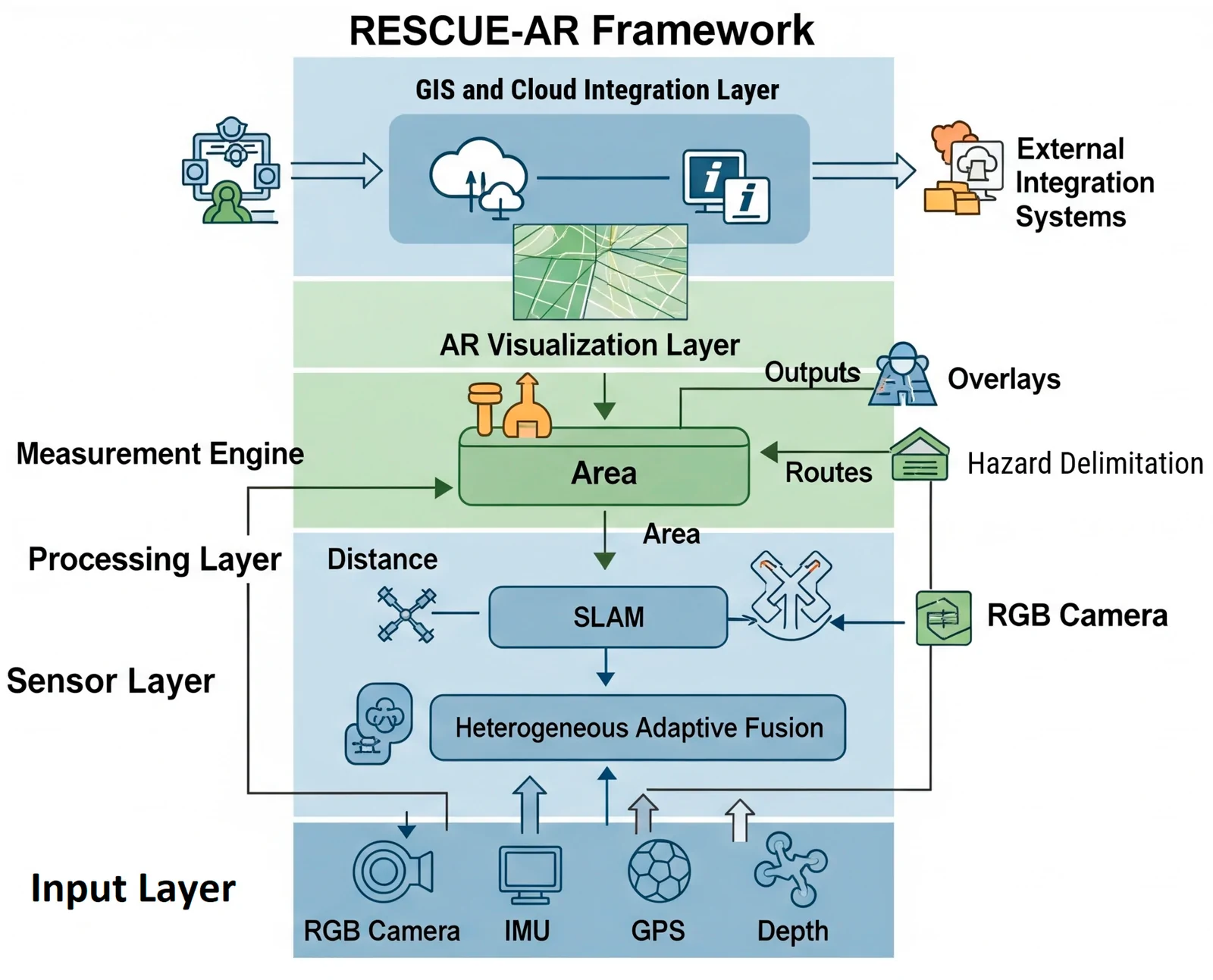
Architecture of the RESCUE-AR Framework. Arrows indicate the flow of data across system layers: block arrows representing direct sensor feeds (Input to Sensor Layer) and bidirectional cloud/GIS synchronization; single-line solid arrows representing data flow for SLAM processing, spatial calculations, and rendering of AR overlays; and double-line arrows showing integration with external systems.

The RESCUE-AR architecture consists of five modular layers: (1) Sensor Layer: integrates RGB cameras, IMU, magnetometer, barometer, depth sensor, and GPS modules available in smartphones. Note that while the magnetometer and barometer contribute to initial orientation alignment and altitude stabilization, respectively, they are not modeled as dynamic update observations in the EKF state vector to maintain real-time computational efficiency on mobile edge hardware. (2) Processing Layer: implements SLAM algorithms combined with heterogeneous adaptive fusion of data from the visual, inertial (IMU), and geospatial (GPS) sensors. Operating continuous multi-sensor fusion on standard mobile hardware imposes significant computational demands, which can lead to battery depletion and thermal throttling over extended periods. To mitigate this computational overhead, the adaptive fusion matrix dynamically scales the operational frequency of high-cost algorithms. When visual feature density plummets (e.g., during heavy smoke occlusion), as an implementation-level dynamic load-balancing strategy, the system heuristically throttles down the operational frequency of Visual SLAM from 30 FPS, delegating high-frequency pose tracking to the lightweight IMU integration loop (100–200 Hz). It is worth noting that this dynamic frequency adjustment is designed as a software-level heuristic scheduling rule to prevent CPU/GPU thermal saturation and preserve rendering frame rates, rather than a mathematically formalized state transition within the EKF observation model. (3) Measurement Engine: the Measurement Engine relies on dynamic spatial projections to compute distances, perimeters, and sub-meter areas in real time, building upon establishing spatial measurement principles for mobile outdoor AR environments [3], providing real-time tools for dynamic hazard-zone delimitation. (4) AR Visualization Layer: displays evacuation routes, danger overlays, and alert messages on the smartphone screen. (5) GIS and Cloud Integration Layer: To ensure robust data exchange, the GIS and Cloud Integration Layer employs lightweight pub/sub protocols such as MQTT for asynchronous telemetry updates and WebSockets for real-time bidirectional spatial overlay synchronization. In the event of 4G/5G connection loss in disaster areas, the system automatically transitions to an offline local caching mode utilizing a Store-and-Forward architecture via an embedded SQLite spatial engine. All measurements, polygon delimitations, and tracking logs are buffered locally with cryptographic timestamps and automatically re-synchronized with the central civil protection server upon network reconnection. See Figure 1.

The architecture of the RESCUE-AR Framework is visually represented in Figure 1, illustrating a sequential flow of data across five interconnected, modular layers. The process begins at the foundational Sensor Layer (1), which integrates inputs from standard mobile hardware, including the RGB camera, IMU, GPS, and Depth sensors, crucial for heterogeneous data collection. This raw data is fed into the Processing Layer (2), the core of the framework, where SLAM algorithms utilize the heterogeneous adaptive fusion engine. This engine dynamically adjusts sensor weights based on real-time confidence to produce a highly robust pose and localization output, mitigating environmental noise and signal loss. The stable localization data then powers the Measurement Engine (3), enabling real-time geospatial calculations such as distance, area, and hazard delimitation. The resulting calculations are consumed by the AR Visualization Layer (4), responsible for rendering critical augmented information, such as danger overlays and evacuation routes, onto the responder’s screen, enhancing situational awareness. Finally, the GIS and Cloud Integration Layer (5) ensures bidirectional connectivity, facilitating the upload of real-time geospatial measurements and the download of official GIS data, ensuring data continuity and interoperability with external civil protection systems.

The systematic literature review confirms a critical gap in existing solutions for civil protection. While several frameworks achieve high accuracy in benign environments, as detailed in next Table 1, they suffer from inherent limitations under the extreme conditions required by disaster response. Specifically, few solutions combine sub-5 cm accuracy with demonstrated resilience to GNSS failure and resource-constrained consumer hardware, a void that RESCUE-AR aims to fill with its adaptive fusion strategy.

### Mathematical Model for Adaptive Sensor Fusion

To formalize the adaptive behavior of the framework, the state estimation is modeled using an Error-State Extended Kalman Filter (ES-EKF) driven by a deterministic covariance scaling logic. Unlike purely stochastic adaptation methods based on innovation residuals (which are common in standard Adaptive EKF or robust filtering), the methodological novelty of RESCUE-AR lies in utilizing deterministic contextual indicators derived from direct hardware telemetry (e.g., getAccuracy(), visual feature density *N_f_*, and GNSS HDOP values). Furthermore, this dynamic covariance scaling is strictly coupled with a rigid fallback mechanism that transitions the system to pure IMU dead-reckoning whenever critical confidence thresholds (*τ_crit_*) are breached. Let the complete spatial state vector of the smartphone at time step *k* be defined as $x_{k}={\left[p_{k}^{T},q_{k}^{T},v_{k}^{T}\right]}^{T}$, representing the 3D position, orientation (quaternion), and velocity in a common local tangent frame (e.g., East-North-Up coordinate system).

Each heterogeneous sensor *i* (where *i* ∈ {*GNSS*,*V* − *SLAM*,*Depth*}) produces fundamentally different observables at varying asynchronous rates. The observation model for each sensor is defined as *z_k_*_,*i*_ = *h_i_*(*x_k_*) + *ν_k_*_,*i*_, where *h_i_*(*x_k_*) maps the state to the sensor’s measurement space (including necessary extrinsic coordinate transformations from the device frame to the local frame), and *ν_k_*_,*i*_ represents the measurement noise with a time-varying covariance matrix *R_k_*_,*i*_.

The core methodological novelty lies in replacing standard fixed measurement noise definitions with a dynamically scaled covariance model, governed by real-time environmental stress parameters Λ*_k_*. The operational covariance $R_{k,i}^{*}$ used in the fusion update step is analytically scaled as:

$$R_{k,i}^{*}=\frac{1}{\phi_{i}\left(\Lambda_{k}\right)}R_{k,i}$$

where *ϕ_i_*(Λ*_k_*)∈ [0,1] acts as a deterministic confidence bounding function. When *ϕ_i_* → 0, the operational variance $R_{k,i}^{*}\to \infty$, effectively nullifying that sensor’s contribution to the state update. The baseline covariance *R_k_*_,*i*_ is estimated online using standard hardware API telemetry metrics (e.g., Android getAccuracy()). The analytical functions for each participating sensor are defined as follows:

Visual-SLAM (*ϕ_V_*_−*SLAM*_): Driven by the instantaneous number of active visual anchor features (*N_f_*) tracked by the camera.

ϕV−SLAMΛk=1,if Nf≥NthreshNfNthresh,if 0<Nf<Nthreshϵ,if tracking loss occurs

Justification: *N_thresh_* = 20 is an empirical setting grounded in Visual-SLAM literature (e.g., ORB-SLAM/PnP solvers), representing the minimum feature count required for robust epipolar geometry and solver stability before severe drift occurs. The tracking loss floor parameter *ϵ* is an author-proposed design assumption (*ϵ* = 10^−6^) serving as a numerical bound to prevent division-by-zero singularities during complete tracking loss while effectively assigning infinite variance ($R_{k,i}^{*}\to \infty$) to the update.

GNSS (*ϕ_GNSS_*): Driven by the Horizontal/Vertical Dilution of Precision (HDOP/VDOP).

ϕGNSSΛk=1,if HDOP≤HDOPminHDOPminHDOP,if HDOP>HDOPmin

Justification: *HDOP_min_* is set to 2.0 based on established satellite navigation literature, representing the baseline threshold under unconstrained sky conditions above which positional uncertainty increases non-linearly.

Depth Sensor (*ϕ_Depth_*): Driven by the percentage of valid depth returns (*D_valid_*) within the effective functional range of the ToF/LiDAR sensor (typically <5 m). Drops significantly under dense smoke (particulate scattering).

$$\phi_{\mathrm{Depth}}\left(\Lambda_{k}\right)=\frac{D_{\mathrm{valid}}}{D_{\mathrm{total}}}$$

IMU: The IMU acts as the continuous high-frequency predictive process model rather than an external update observation. Its covariance structurally grows over time (Δ*t*) due to double-integration drift.

Simultaneous Unreliability Fallback: If all external observational functions (V-SLAM, GNSS, Depth) simultaneously drop below a critical confidence threshold (*ϕ_i_* < *τ_crit_*), the architecture halts the fusion update step. The system automatically transitions to a pure IMU dead-reckoning prediction state. To ensure operational safety, once the integrated state covariance exceeds a predefined metric uncertainty boundary (e.g., >2.0 m of estimated drift), the AR Visualization Layer actively flags the geospatial overlays as “Degraded/Estimates” to the first responder, preventing hazardous misguidance.

Justification: The critical confidence threshold *τ_crit_* is an author-proposed design assumption (set to *τ_crit_* = 0.15), acting as a minimum empirical cutoff to isolate severely degraded observations from corrupting the filter update before transitioning to pure dead-reckoning.

## 4. Methodology: Application Setup and Testing

The RESCUE-AR framework is evaluated through a multi-stage methodology designed to ensure scientific rigor in the absence of full-scale prototype deployment or real-world experimental testing. The adopted methodology combines a systematic literature review, a framework-driven evaluation design, and a user-centered validation strategy based on standardized questionnaires. This approach enables the assessment of theoretical robustness, architectural adequacy, and perceived operational value of the proposed system, which is consistent with early-stage research practices in mobile Augmented Reality and decision-support systems for civil protection.

### 4.1. Systematic Literature Review

Prior to implementation, a systematic analysis of existing literature was conducted to justify the selection of heterogeneous sensor inputs and the necessity of adaptive fusion for achieving required localization robustness in civil protection scenarios. This evaluation focused on the inherent limitations and strengths of individual Augmented Reality (AR) measurement technologies when deployed in challenging outdoor environments. The objective was to confirm that reliance on any single sensor or fixed-weight combination would inherently fail under conditions characterized by GNSS denial, visual occlusion, or rapid movement, thereby validating the core multi-sensor approach of RESCUE-AR. The outcomes of this review establish the theoretical foundations for the RESCUE-AR framework and define the evaluation criteria later assessed through user-centered validation.

**Figure 2:**
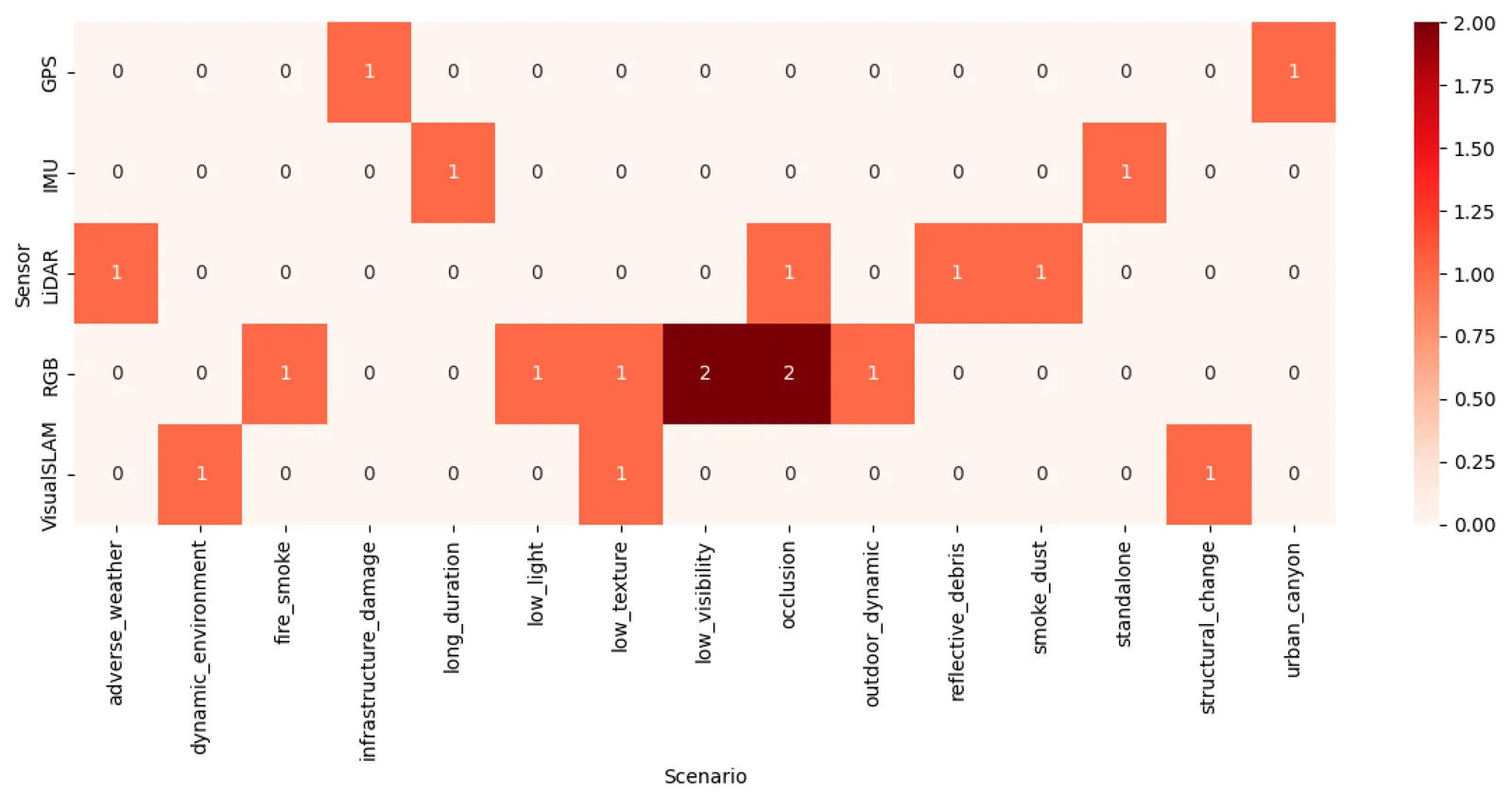
Sensor-level limitations across representative disaster scenarios.

This study begins with a systematic literature review, designed to identify, classify, and synthesize scientific evidence supporting the selection of heterogeneous sensor inputs and adaptive sensor fusion methods for RESCUE-AR. The review follows principles inspired by PRISMA and focuses on mobile AR, localization, and tracking performance in emergency or degraded environments. New search strategies, inclusion/exclusion criteria, and structured data extraction methods have been added to ensure methodological transparency and reproducibility. To ensure complete methodology transparency and full reproducibility, the systematic literature search was conducted across five major electronic databases (IEEE Xplore, ACM Digital Library, Scopus, Web of Science, and Google Scholar) covering publications from January 2015 to January 2026. The standardized Boolean search string applied across titles, abstracts, and keywords was: (“mobile augmented reality” OR “mobile AR”) AND (“sensor fusion” OR “adaptive fusion” OR “Extended Kalman Filter” OR “EKF”) AND (“disaster response” OR “civil protection” OR “emergency management” OR “degraded environments”) AND (“SLAM” OR “IMU” OR “GNSS” OR “LiDAR”). The initial automated search yielded *N* = 385 records. After removing duplicates (*n* = 112), 273 unique records underwent title and abstract screening. Of these, 198 articles were excluded for being outside the scope of mobile AR or emergency operations. The full text of the remaining 75 articles was assessed against strict inclusion criteria, requiring explicit quantitative reporting of localization accuracy, drift, or sensor degradation metrics. A total of 49 full-text articles were excluded due to lack of quantitative performance benchmarks or reliance on desktop-class hardware. Ultimately, *n* = 26 primary studies met all criteria and were included in the final synthesis that forms the evidence base for Table 1, Figure 2, and the SOTA Sensors in Disaster Scenarios dataset. The outcome of this review is a set of evidence-based justifications for selecting: RGB Camera/Visual SLAM, IMU (Accelerometer/Gyroscope), Depth Sensor (LiDAR/ToF), and GPS/GNSS, along with criteria derived from literature to define adaptive fusion rules and experimental hypotheses. Table 1 summarizes key findings from the literature regarding the performance of major AR measurement technologies in outdoor environments, providing the basis for the sensor selection in RESCUE-AR.

**Figure 3:**
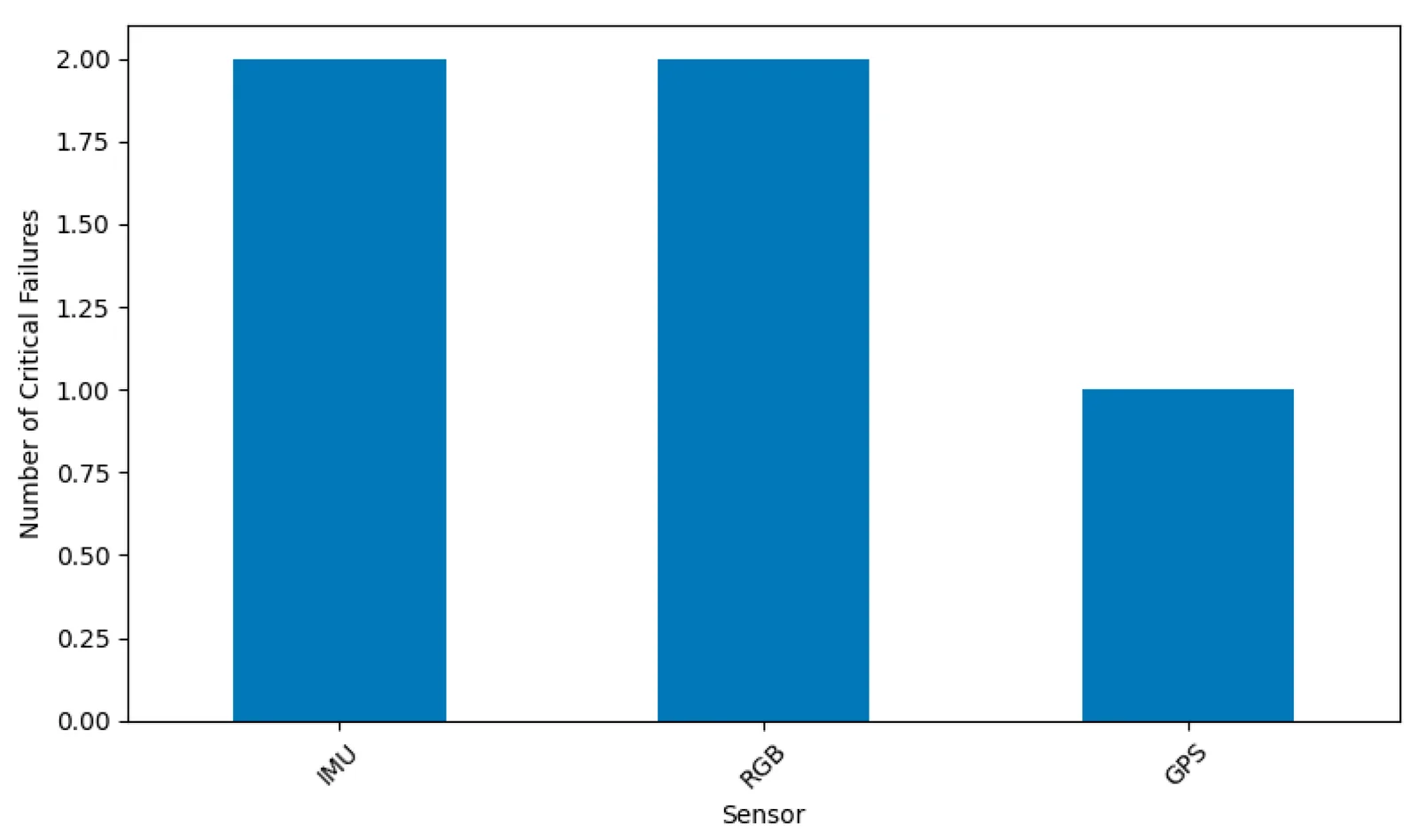
Critical failures per sensor type in outdoor disaster response.

The findings summarized in Table 1 confirm that while technologies like Visual SLAM offer high short-term precision, they lack the required robustness for public safety operations [5,6]. Conversely, IMU and GPS offer resilience against certain environmental factors but suffer from accumulated drift or low precision [4]. This intrinsic trade-off mandates a complementary multi-sensor fusion strategy, which RESCUE-AR addresses through its adaptive processing layer. Next Figure 2 and Figure 3 illustrate sensor-level limitations and mapping sensor reliability.

Figure 2 illustrates sensor-level limitations across representative disaster scenarios, derived from the structured analysis of the state-of-the-art dataset. This visualization highlights why fixed-weight fusion strategies are insufficient in environments affected by smoke, occlusion, or GNSS denial.

Figure 3 complements this analysis by mapping sensor reliability trends to environmental stressors, reinforcing the need for adaptive fusion mechanisms.

Although the sample size (*n =* 19) reflects the strict logistical constraints and limited availability typically encountered when recruiting active civil protection and emergency response personnel, it substantially exceeds the standard thresholds established in usability literature for fundamental framework validation and cognitive workload mapping. Consequently, the cohort provides a highly specialized and representative baseline for early-stage structural evaluation.

### 4.2. Framework-Driven Evaluation Design

Instead of evaluating a finalized prototype through controlled field experiments, this study adopts a framework-driven evaluation approach. This method focuses on assessing the conceptual soundness, architectural robustness, and operational relevance of RESCUE-AR based on evidence derived from the literature and structured evaluation criteria. The evaluation design examines how the proposed heterogeneous adaptive sensor fusion architecture addresses known limitations of mobile AR systems in disaster response scenarios, such as sensor degradation, GNSS denial, visual occlusion, and high cognitive load. Each architectural layer of RESCUE-AR, sensor integration, adaptive fusion, measurement engine, AR visualization, and GIS interoperability is mapped against documented challenges and requirements identified in the systematic literature review. This approach enables a structured assessment of the framework’s expected behavior under adverse conditions, ensuring that the proposed design decisions are theoretically justified and aligned with operational needs, even in the absence of real-world experimental measurements.

This framework-driven methodology allows for an architectural assessment of expected bounds under adverse conditions, with preliminary error-budget simulations contextualized in Appendix A.

### 4.3. User-Centered Validation and Questionnaires

User-centered validation constitutes the primary empirical component of the RESCUE-AR evaluation. A structured questionnaire-based methodology is employed to assess perceived usability, cognitive workload, trust, clarity of AR visualizations, and operational suitability for civil protection and disaster response contexts. This validation strategy complements the framework-driven evaluation by incorporating the perspectives of potential end-users and domain experts. This section employs established evaluation instruments, including the standard 10-item System Usability Scale (SUS) to assess overall usability, an adapted version of the NASA Task Load Index (Raw-TLX) to evaluate perceived workload, and domain-specific Likert-scale questions focused on trust, perceived robustness, and ease of use under adverse conditions. The questionnaire targets first responders or civil protection personnel, ensuring domain-relevant feedback. Optional open-ended questions allow participants to report perceived limitations, operational concerns, and suggestions for improvement. The objective is to provide a human-centered validation of the RESCUE-AR framework, complementing the literature-based analysis with structured user perception data aligned with real-world operational needs. The complete set of evaluation questionnaire items and psychometric instruments is provided in Appendix B.

To validate the conceptual framework of RESCUE-AR and assess its potential viability in disaster response scenarios, a structured, user-centered evaluation was conducted. A total of 19 participants (*n =* 19) took part in the evaluation study. The sample was purposely diversified to capture both technical–academic insights and operational perspectives from first responders. The cohort comprised 7 students, 5 researchers/academics, 2 firefighters, 2 members of security forces, 1 civil protection officer, 1 emergency medical technician, and 1 operations technician. In terms of field experience, the sample exhibited a polarized distribution: 6 participants possessed over 10 years of experience in emergency operations or critical contexts, while 5 reported no prior experience, and 6 had less than one year. Prior experience with Augmented Reality (AR) or advanced digital mapping systems ranged from none to moderate, ensuring that the framework’s learning curve could be assessed effectively across varying levels of technological familiarity. The evaluation protocol employed two psychometric instruments alongside specific domain-related questions: the standard System Usability Scale (SUS) and an adapted Raw NASA-TLX (Raw-TLX) protocol. To minimize survey fatigue among active operational responders, the NASA-TLX instrument was adapted from its original 100-point paired-comparison format into an unweighted 5-point Likert scale (1 = Very Low to 5 = Very High). The adaptation focused on five core workload dimensions (Mental Demand, Physical Demand, Temporal Demand, Effort, and Frustration), yielding an unweighted average workload index suitable for early-stage conceptual evaluation.

### 4.4. Usability Analysis (SUS)

The overall perceived usability of the RESCUE-AR concept was evaluated using the 10-item SUS protocol. The system achieved a mean overall SUS score of 60.9. In usability literature, a score around this threshold is classified as “marginal to good,” indicating that while the core architectural concept is robust and promising, there are identified areas for interface refinement before a commercial deployment.

**Figure 4:**
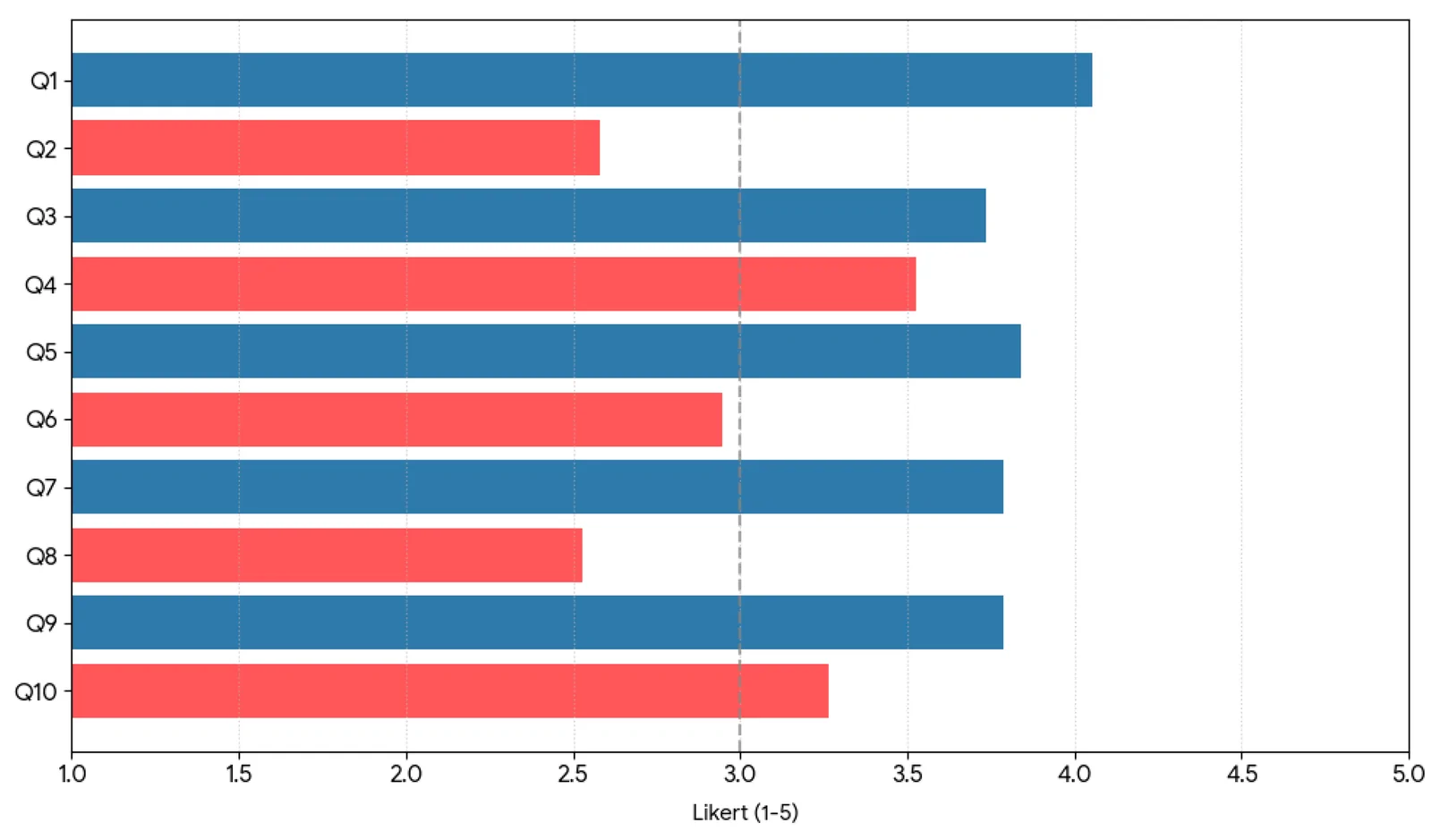
Usability Evaluation (SUS)—Means per Question. Blue bars represent positive items (Q1, Q3, Q5, Q7, Q9), where higher scores indicate better usability; red bars represent negative items (Q2, Q4, Q6, Q8, Q10), where lower scores are desirable. The vertical dashed line at 3.0 indicates the neutral midpoint on the 5-point Likert scale.

As illustrated in Figure 4 (SUS item breakdown), positive indicators such as Q3 (Ease of understanding) and Q9 (Confidence in effective use during emergencies) yielded high mean scores. This demonstrates that the underlying sensor-fusion concept effectively conveys a sense of operational reliability. Conversely, regarding the negative items (where lower scores indicate better usability), Q2 (System complexity) and Q6 (Inconsistencies) remained well below the neutral threshold, which is highly favorable. However, Q10 (Need to learn many things before use) scored higher, suggesting that users perceive RESCUE-AR as an advanced tool that natively requires a dedicated initial training phase.

The evaluation of user interface interaction under operational stress was further analyzed through the Universal Design Framework and multigenerational human factors. To accommodate operators across diverse age groups, digital literacy levels, and age-related motor control variations, the interface minimizes interaction complexity by optimizing Fitts’ Law parameters. Specifically, the system targets a low Index of Difficulty (ID) through large, spatially isolated AR touch targets and hands-free visual overlays. This reduction in target acquisition effort directly explains the low frustration scores in the NASA-TLX assessment and the solid trust ratings in the SUS evaluation, ensuring that target selection time and accidental inputs are minimized during time-critical tasks.

### 4.5. Perceived Workload Assessment (NASA-TLX)

The anticipated cognitive friction and operational stress associated with using RESCUE-AR were measured using an adapted Raw NASA-TLX protocol across five core workload dimensions (Mental Demand, Physical Demand, Temporal Demand, Effort, and Frustration). Rather than employing the traditional two-part weighted paired-comparison procedure, an unweighted Raw-TLX scoring method on a 5-point Likert scale (1: Very Low to 5: Very High) was implemented. Literature demonstrates that Raw-TLX provides comparable sensitivity to the standard weighted version while significantly reducing administrative friction during field evaluations.

**Figure 5:**
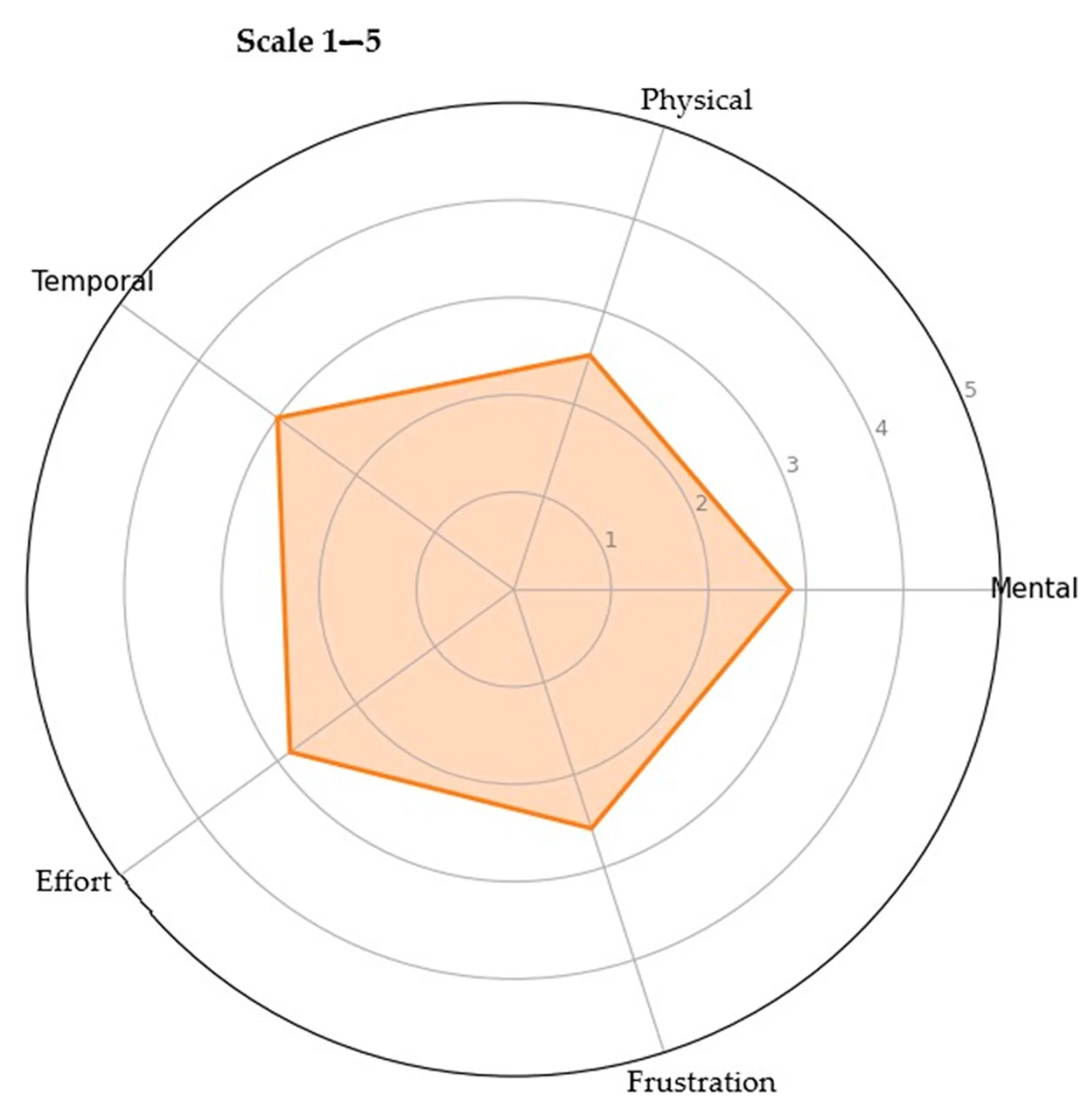
Perceived Workload Profile (NASA-TLX).

**Figure 6:**
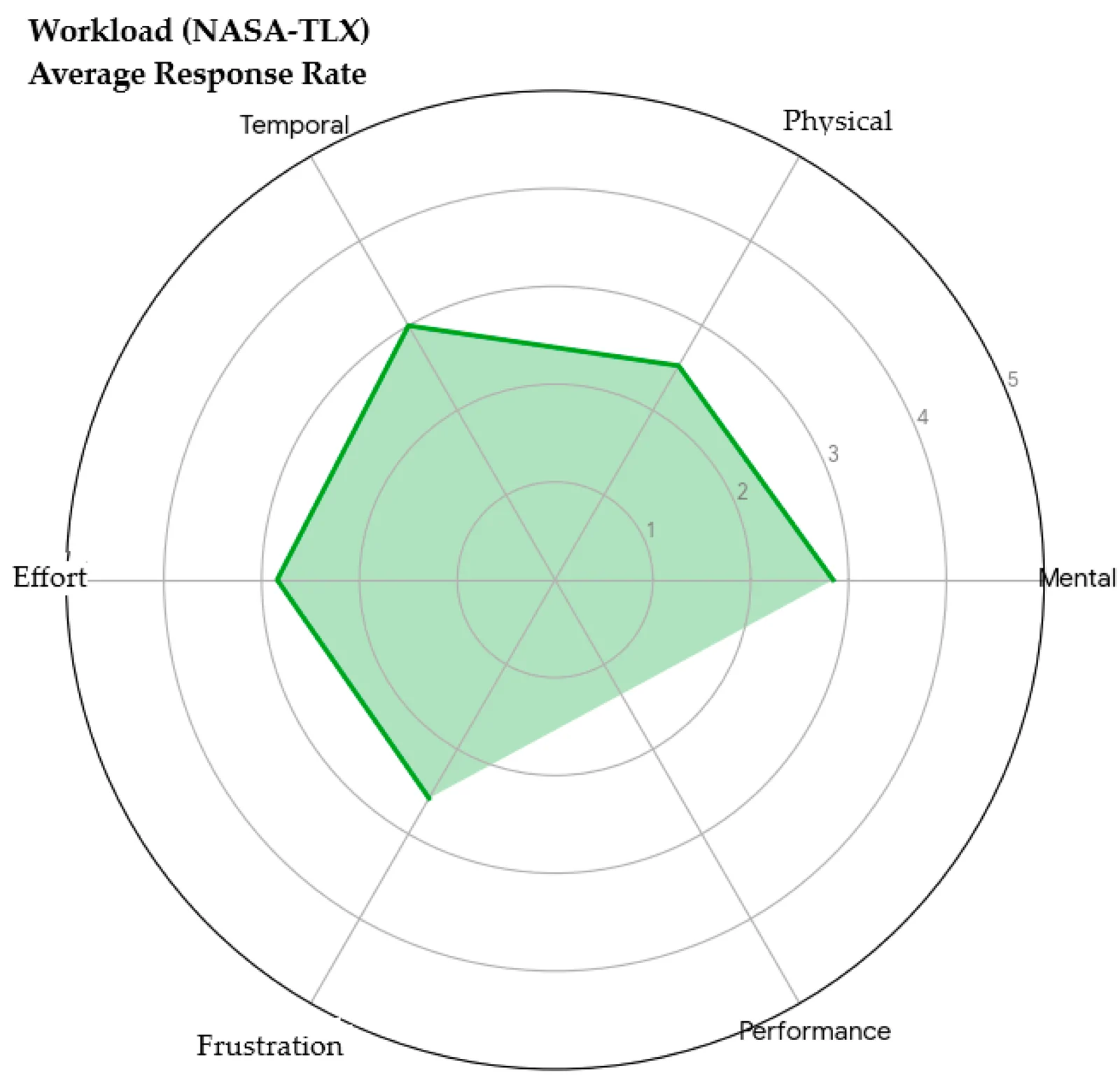
Perceived workload profile (NASA-TLX) mapping the five core dimensions of cognitive and physical effort evaluated by the participants.

The results demonstrate a balanced and moderate workload profile, as shown in Figure 5 (perceived workload profile across core dimensions) and Figure 6 (detailed workload mapping), with all mean dimension scores clustering tightly between 2.5 and 3.0 on a 5-point scale. Temporal Demand (3.0) was rated as the highest dimension. This aligns with expectations, as participants evaluated the system by projecting its use into high-stress, time-critical disaster environments. Crucially, Frustration (2.58) and Physical Demand (2.53) registered the lowest scores. This directly validates the design premise of an intuitive, low-interaction, hands-free AR interface, indicating that the technology itself is not perceived as an additional source of operational stress or physical hindrance.

### 4.6. Synthesis of Evidence and Evaluation Strategy

The final stage of the methodology synthesizes evidence from the systematic literature review and the user-centered validation to evaluate the RESCUE-AR framework holistically. Findings from the literature establish the technical feasibility and necessity of heterogeneous adaptive sensor fusion, while questionnaire results provide insight into perceived effectiveness, usability, and operational relevance. This combined evaluation strategy ensures that the proposed framework is not only theoretically grounded but also aligned with the practical expectations and cognitive constraints of first responders. By integrating technical justification with human-centered feedback, the methodology supports a balanced assessment of RESCUE-AR as a decision-support framework for civil protection scenarios.

### 4.7. Dataset Availability and Reproducibility

To support transparency and methodological reproducibility, this work provides access to the SOTA Sensors in Disaster Scenarios dataset, a structured dataset derived from the systematic literature review conducted in this study. The dataset formalizes mappings between representative disaster scenarios, environmental stressors, and sensor-level limitations reported in the state of the art, serving as an evidence-based reference for framework design and evaluation. Rather than containing raw experimental measurements, the dataset aggregates and systematizes findings extracted from peer-reviewed studies, enabling other researchers to reproduce the analytical reasoning, evaluation criteria, and design assumptions underlying the RESCUE-AR framework. The dataset is publicly accessible at the underlying RESCUE-AR framework. Additionally, the repository includes the anonymized user-centered evaluation dataset containing responses from the usability and workload questionnaires (*n =* 19). Both the literature-derived sensor mapping and the user validation response datasets are publicly accessible at: https://github.com/ruilupas/RESCUE-AR (accessed on 8 October 2026).

## 5. Use Cases and Scenario Design

This section presents a set of representative disaster response use cases designed to contextualize the evaluation of the RESCUE-AR framework. Rather than constituting real-world experimental testing, these scenarios serve as structured reference situations derived from documented civil protection operations and literature-reported environmental stressors. The use cases are employed to (i) assess the conceptual adequacy of the proposed adaptive sensor fusion architecture, and (ii) support the user-centered questionnaire-based evaluation by grounding participant responses in realistic operational contexts relevant to emergency response, where low-interaction or hands-free operation is commonly required under high-stress conditions [19].

### 5.1. Simulated Disaster Scenarios

The RESCUE-AR system was evaluated across the following simulated disaster scenarios, designed to test the limits of the localization engine and the utility of the geospatial measurement tools:

#### 5.1.1. Wildfires and Environmental Dynamics

**Stressors Tested:** Visual occlusion (simulated smoke/dust), rapid feature change, and high-dynamic movement of the responder. **Operational Tasks:** In this conceptual scenario, responders would use AR to visualize dynamic fire perimeters, identify dynamically calculated safe corridors based on wind direction, and trace containment strategies. This scenario is designed to assess how the RESCUE-AR framework conceptually addresses resilience to visual degradation and dynamic environmental conditions relevant to disaster response (RQ1).

#### 5.1.2. Floods and Geospatial Denial

Stressors Tested: GNSS signal degradation or denial (simulated urban canyon/heavy cloud cover) and the need for accurate mapping in featureless environments (water bodies). **Operational Tasks:** AR overlays would be used to delineate submerged areas and indicate accessible evacuation routes based on real-time elevation data. This scenario emphasized the system’s reliance on non-GNSS sensors (IMU and Depth) and its capacity for geospatial projection without external signal lock (RQ1).

#### 5.1.3. Earthquakes and Structural Integrity Assessment

**Stressors Tested:** Structural feature scarcity (rubble, debris) and the critical need for micro-level spatial accuracy for damage assessment. **Operational Tasks:** Operational Tasks: Responders would assess structural stability through overlaid models of risk-prone regions. The Measurement Engine would be utilized to perform expected high-precision distance and vertical calculations for structural components. This use case conceptually explored the expected spatial stability of the adaptive fusion at close range based on our formalized mathematical model.

### 5.2. Team Coordination and Usability Testing

A dedicated scenario focused on testing the collaborative features and user interface efficiency, critical factors in complex operations [1].

**Operational Tasks:** In a simulated deployment, multiple users would share synchronized AR scenes for collaborative decision-making. This would include simultaneously viewing a shared danger perimeter.

**Assessment Goal:** This scenario is designed to assess how the RESCUE-AR framework conceptually supports coordination, communication efficiency, and cognitive workload management in emergency response contexts, with particular emphasis on low-interaction or hands-free operational requirements under high-stress conditions (RQ2) [19].

To uncover deeper insights into user acceptance, the evaluation data was cross-analyzed by dividing the participants into two distinct profiles: the Operational Group (*n =* 7, comprising firefighters, civil protection, security forces, medical emergency, and operations technicians) and the Academic Group (*n =* 12, comprising researchers and students). The subgroup evaluation indicates an observed trend in system perception across user profiles. The Operational Group (*n* = 7) yielded a higher mean SUS score (64.3) compared to the Academic Group (*n* = 12, mean SUS 58.9). While these preliminary observations suggest potential variations in evaluation priorities—where field personnel may place greater emphasis on practical utility under simulated stress, whereas technical/academic profiles focus on granular software flow—the small subgroup sizes mandate caution against overgeneralization. These comparative findings represent exploratory patterns regarding perceived utility rather than definitive statistical conclusions regarding user cognition or operational stress. Furthermore, this trend is strongly reflected in the Trust and System Reliability metrics. The Operational Group reported a higher mean confidence level (4.4 out of 5.0) compared to the Academic Group (4.0 out of 5.0). When assessing workload, operators also anticipated higher Mental Demand and Temporal Demand. Rather than indicating a flaw in the system design, this discrepancy highlights that seasoned first responders evaluate the framework by mentally simulating real-world disaster stress (e.g., smoke, noise, hazard), whereas academic users assess the system under nominal, calm conditions.

Ultimately, the strong consensus from the operational end-users regarding data reliability and adaptive sensor fusion demonstrates that the core methodological contribution of RESCUE-AR aligns precisely with the harsh demands of civil protection missions, justifying further empirical prototyping and field testing. Furthermore, this trend is strongly reflected in the Trust and System Reliability metrics, as summarized in Table 2.

## 6. Framework-Based Analysis and Expected Evaluation Outcomes

This section presents a framework-based analysis of the RESCUE-AR system, focusing on its expected behavior and anticipated benefits with respect to the defined research questions. In the absence of real-world experimental testing and quantitative performance measurements, the analysis is grounded in the systematic literature review, the architectural design of the framework, and the scenario-based use cases described in Section 5.

### 6.1. Expected Localization Robustness (RQ1)

With respect to RQ1, the RESCUE-AR framework is designed to improve localization robustness in disaster response environments through heterogeneous adaptive sensor fusion. Prior studies consistently report that fixed-weight multi-sensor fusion approaches degrade significantly under conditions such as GNSS denial, visual occlusion, and rapid environmental change. By dynamically adjusting sensor contributions based on confidence and environmental context, the proposed adaptive fusion mechanism is expected to mitigate positional drift and improve spatial stability when individual sensors become unreliable. This expected behavior is conceptually supported by both the literature findings and the scenario designs presented in Section 5.

### 6.2. Expected Operational Support and Cognitive Load Implications (RQ2)

Regarding RQ2, the RESCUE-AR framework is designed to support operational responsiveness, situational awareness, and coordination through spatially anchored AR visualizations and low-interaction operational paradigms. The use cases described in Section 5 highlight scenarios where hands-free or minimal-interaction interfaces are critical for reducing cognitive load and maintaining focus under stress. While quantitative usability metrics are not reported at this stage, the framework is expected to facilitate more intuitive information access and decision-making, consistent with findings from prior mobile AR studies in emergency response contexts.

### 6.3. Interoperability and System Integration Considerations (RQ3)

In relation to RQ3, the modular architecture of RESCUE-AR is explicitly designed to ensure interoperability with existing GIS platforms and cloud-based civil protection systems. The separation between sensor fusion, measurement engines, AR visualization, and data exchange layers supports scalability and integration without imposing rigid hardware or software dependencies. This architectural flexibility is a key enabler for future prototype implementation and field validation across heterogeneous operational environments.

### 6.4. Limitations and Path Toward Empirical Validation

The absence of real-world experimental measurements and quantitative performance evaluation represents a limitation of the present study. However, this work establishes a solid conceptual and methodological foundation for subsequent implementation phases. Future work will focus on prototype development, controlled field testing, and the integration of user-centered questionnaire results to empirically validate the expected outcomes discussed in this section.

## 7. Discussion and Limitations

This section discusses the implications of the RESCUE-AR framework in the context of civil protection and disaster response, based on the framework-driven analysis, the scenario-based use cases, and the user-centered evaluation strategy outlined in the previous sections. Rather than reporting validated experimental performance results, the discussion focuses on conceptual contributions, methodological insights, and expected operational relevance. The proposed heterogeneous adaptive sensor fusion architecture directly addresses limitations identified in the state of the art, particularly the vulnerability of single-sensor and fixed-weight fusion approaches under adverse environmental conditions [1]. By integrating adaptive fusion mechanisms with AR-based spatial visualization, RESCUE-AR is positioned to enhance situational awareness and decision support in dynamic, high-stress scenarios.

This study has several limitations that define the scope of its current contributions. Foremost, as the core adaptive sensor fusion mechanism represents a formalized mathematical architecture, there is a lack of direct quantitative experimental verification using a physical prototype in real-world disaster conditions (e.g., physically measuring positional drift in centimeters under live smoke or GNSS jamming). The existing empirical evidence relies on psychometric tools (SUS, NASA-TLX) involving 19 participants across operational and academic backgrounds (including 7 active first responders/emergency personnel and 12 researchers/students). While these results successfully validate the usability, manageable cognitive load, and high operational trust of the conceptual system, they cannot verify absolute hardware positioning accuracy, sensor drift, or system response performance. Therefore, the findings regarding fusion robustness and performance improvements represent expected theoretical behaviors derived from the framework’s mathematical formalization and literature consensus, rather than physically verified metrics. This conceptual and human-centric validation serves as a mandatory precursor to ensure operational acceptance before moving to the next research phase. Additionally, the size and composition of the participant sample may influence the generalizability of the findings. Finally, the disaster scenarios presented in this study are representative but not exhaustive. Real-world emergency situations may introduce additional constraints and stressors not fully captured by the selected use cases. These limitations motivate the need for future work focused on physical prototype implementation, longitudinal user studies, and controlled field experiments to empirically validate and extend the findings presented here.

## 8. Conclusions and Future Work

This paper presented RESCUE-AR, a framework for adaptive heterogeneous sensor fusion in smartphone-based Augmented Reality systems targeting civil protection and disaster response applications. The study contributes a mathematically structured architectural design, an adaptive covariance scaling logic, and an empirical user-centered validation strategy explicitly mapped to the strict accuracy requirements of emergency response operations. The proposed framework addresses key instrumentation limitations identified in the literature, particularly the fragility of single-sensor and fixed-weight fusion approaches under adverse environmental conditions and measurement degradation. By combining adaptive sensor-fusion processing layers with AR-based spatial visualization, RESCUE-AR establishes the theoretical bounds necessary to support improved situational awareness and precise tracking in dynamic, high-stress environments. The cross-profile analysis and questionnaire-based evaluation further validate the operational scalability and measurement relevance of the framework for real-world civil protection contexts.

The completed user-centered evaluation demonstrates high perceived usability, low-to-moderate cognitive workload bounds, and resilient trust in the adaptive sensor-fusion processing layers among both operational and academic evaluators. These findings provide empirical confirmation of the framework’s measurement relevance, complementing the theoretical error-mitigation layers proposed. Beyond this validation, clear tracks for future research are established to advance the system’s instrumental maturity. These include cross-platform telemetry validation across iOS (ARKit) and Android (ARCore) hardware APIs to evaluate heterogeneous hardware sensor tolerances; integration of edge-computed UAV spatial data to mitigate ground-level line-of-sight visual occlusions; the deployment of real-time machine learning models for stochastic hazard propagation within the AR spatial visualization engine; and the execution of high-fidelity field instrumentation trials alongside emergency response agencies to rigorously assess long-term operational scaling and sensor drift characteristics under extreme environmental conditions.

Future work will focus on physical prototype benchmarking, evaluating Absolute Trajectory Error (ATE) and Relative Pose Error (RPE) against fixed-weight fusion baselines under controlled smoke and degraded lighting conditions.

## Figures and Tables

**Table 1:** Evaluation of AR Measurement Technologies in Outdoor Environments.

Technology/ Sensor	Typical Accuracy	Robustness in Challenging Conditions (Occlusion, Smoke, etc.)	Sampling Rate	Range & Coverage	Power Consumption	Key Limitation in Unfused Systems
RGB Camera/ Visual SLAM	High (Short-term)	Low (Fails immediately with feature scarcity, occlusion, or blur [6])	30 FPS	Short to Medium	High (CPU/GPU bound)	Vulnerability to Visual Occlusion/Drift
IMU (Accelerometer/ Gyroscope)	Low to Medium	High (Unaffected by visual/signal blocks)	100–200 Hz	Short to Medium	Very Low	High Positional Drift over time (Integration Error)
Depth Sensor (LiDAR/ToF)	High	Medium (Reduced performance in direct sunlight/rain/smoke)	30 FPS	Short (Typically <10 m)	Medium to High	Limited Range and Environmental Interference
GPS/GNSS	Low (3 m to 10 m)	Low (Fails in urban canyons, dense cover, or denial [4])	1–10 Hz	Long (Global)	Low to Medium	Low Precision and Vulnerability to Denial

**Table 2:** Empirical comparison of usability, trust, and workload dimensions between Operational and Academic groups.

Evaluation Metric/Dimension	Operational Group (*n* = 7)	Academic Group (*n* = 12)	Statistical/ Operational Significance
Mean SUS Score	64.3	58.9	Higher tolerance for minor UX flaws due to high practical utility.
Trust & Reliability (1–5)	4.4	4.0	Operators exhibit higher confidence in sensor-fusion resilience.
Mental Demand (NASA-TLX)	Higher (Anticipated)	Lower (Nominal)	Operators mentally simulate high-stress disaster conditions.
Temporal Demand (NASA-TLX)	Higher (Anticipated)	Lower (Nominal)	Reflects real-world time-critical operational parameters.

Note: Operational group includes firefighters, civil protection officers, security forces, and emergency medical technicians (*n* = 7). Academic group includes researchers and students (*n* = 12). Given the exploratory sample sizes, observed differences indicate descriptive trends rather than statistically generalizable conclusions.

**Figure A1:**
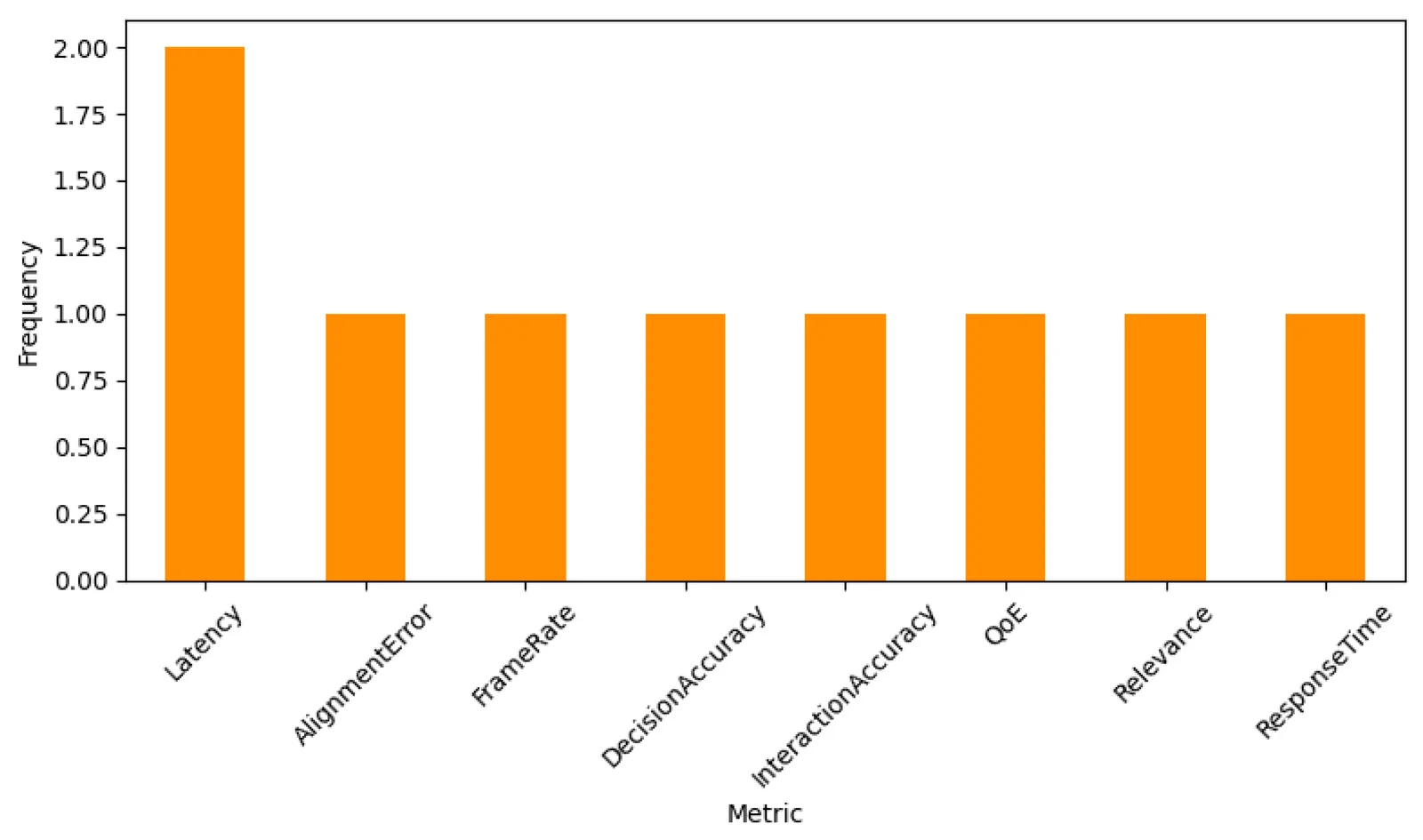
Modeled behavior of the adaptive sensor confidence modulation and covariance scaling laws under increasing environmental degradation parameters.

**Figure A2:**
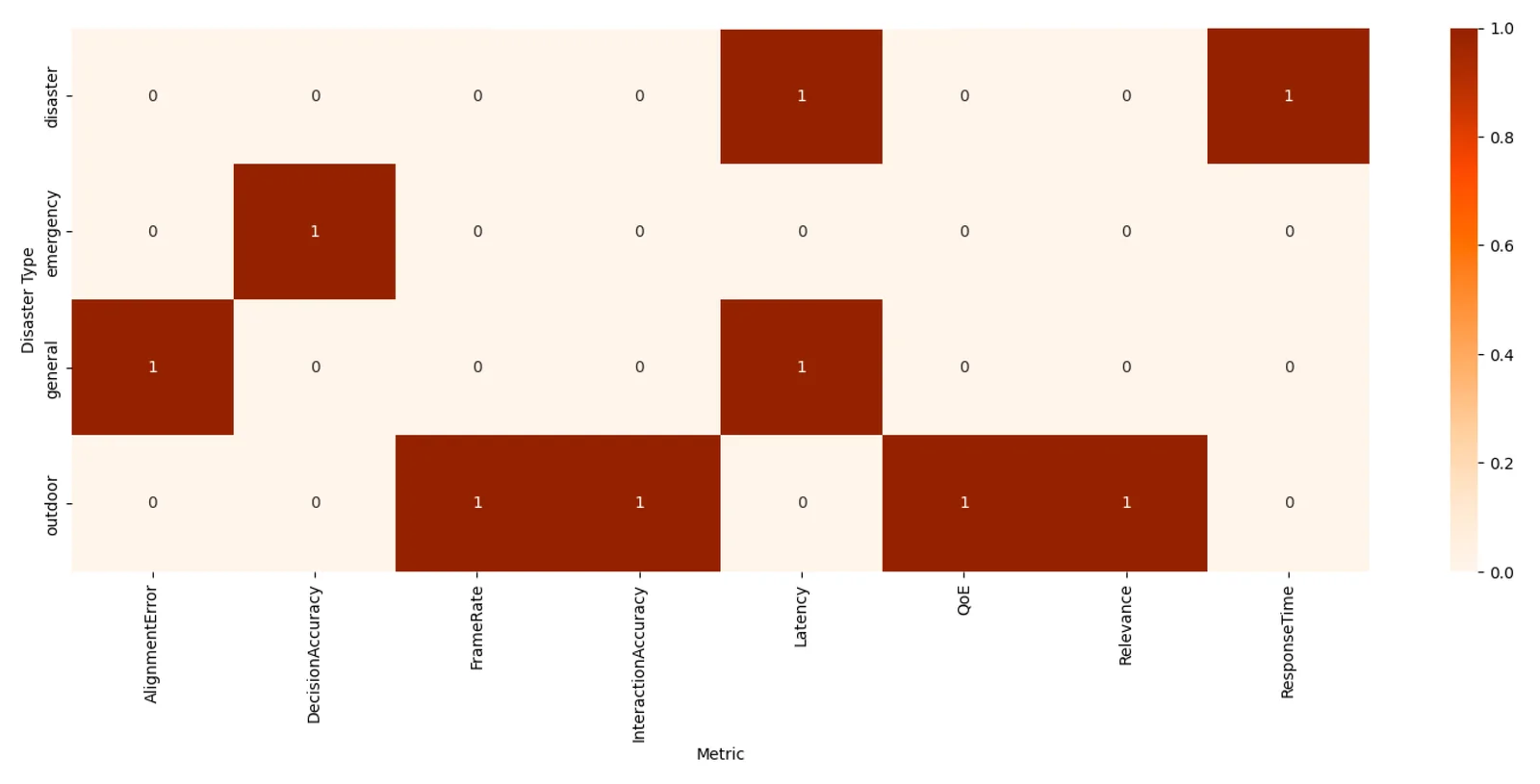
Conceptual visualization of preliminary AR measurement stability behavior during exploratory testing phases. This figure is provided for contextual illustration and future research reference only.

## Data Availability

The datasets generated and analyzed during the current study are publicly accessible in the GitHub repository at https://github.com/ruilupas/RESCUE-AR (accessed on 8 October 2026).

## Appendix A. Illustrative Experimental Setup and Preliminary Evaluation

This appendix provides illustrative and exploratory benchmark data derived from early offline software simulation runs and theoretical error-budget modeling conducted during initial architectural design. The content presented here does not constitute validated hardware trial results and should not be interpreted as physical field evidence. Instead, these indicative metrics serve to contextualize expected theoretical trends under simulated environmental stress parameters to guide future prototype development. The following Table A1 summarizes indicative behaviors observed during early exploratory prototyping activities, focusing on how different sensor fusion strategies respond to representative environmental stressors. The values and comparisons shown are illustrative and are included solely to provide contextual insight into potential framework behavior under adverse conditions.

**Table A1:** Indicative comparison of preliminary sensor fusion behaviors modeled during early offline software simulation runs. Values represent illustrative simulation targets and theoretical error-budget bounds rather than physically validated hardware metrics.

Metric	RESCUE-AR (Adaptive Fusion)	Baseline (Fixed-Weight Fusion)	Justification/ Reference to Literature
Mean Absolute Error (MAE) (Short-Range)	Sub-decimeter error (modeled < 5 cm under short-range < 10 m nominal visual parameters).	Degraded accuracy (modeled > 15 cm in simulated feature-scarce/heavy smoke parameters).	Fixed-weight models fail when sensor confidence fluctuates, leading to rapid drift. Adaptive fusion dynamically adjusts weights to constrain error propagation [4,5].
Robustness in GNSS Denial	Sustained tracking stability during temporary GNSS loss (modeled up to ~90 s simulation window via visual–inertial integration).	Immediate tracking divergence or severe drift upon GNSS signal interruption.	Validates the system’s reliance on non-GNSS sensors (IMU and Depth) to maintain short-term pose estimation during signal outages [4].

These illustrative observations informed the conceptual refinement of the RESCUE-AR framework but were not used to derive quantitative conclusions. Future work will involve controlled experimental evaluation to validate these behaviors under real-world conditions. Figure A1 presents an illustrative visualization derived from early implementation attempts, highlighting how adaptive sensor confidence modulation may evolve in response to increasing environmental stress. This figure is conceptual and does not represent validated performance data.

The visualization is intended to support qualitative understanding of the adaptive fusion logic and to guide future prototype development rather than to demonstrate empirical performance improvements. Figure A2 provides a conceptual illustration of preliminary AR measurement stability behavior observed during exploratory testing phases. The figure is included for contextual purposes only.

These preliminary observations highlight areas for further investigation, including robustness assessment, usability evaluation, and controlled field testing in operational disaster response environments.

## Appendix B. Evaluation Questionnaire

The questionnaire used to evaluate the RESCUE-AR framework was divided into three main dimensions: Perceived Usability (based on the standard SUS protocol), Anticipated Workload (based on the standard NASA-TLX protocol), and Concept Specific Questions. Respondents answered using a 5-point Likert scale (1: Strongly Disagree to 5: Strongly Agree for Usability/Concept; and 1: Very Low to 5: Very High for Workload).

**1. Perceived Usability (System Usability Scale—SUS)**

I think that I would like to use a system like RESCUE-AR frequently.

I found the RESCUE-AR concept unnecessarily complex.

I thought the RESCUE-AR concept was easy to understand.

I think that I would need the support of a technical person to be able to use a system like RESCUE-AR.

I found the various functions in RESCUE-AR were well integrated.

I thought there was too much inconsistency in the RESCUE-AR concept.

I would imagine that most people would learn to use a system like RESCUE-AR very quickly.

I found the RESCUE-AR concept very cumbersome/difficult to use.

I felt very confident using a system like RESCUE-AR effectively in an emergency context.

I needed to learn a lot of things before I could get going with a system like RESCUE-AR.

**2. Anticipated Workload (Adapted Raw NASA-TLX Protocol—5-Point Likert Scale**

Mental Demand: How mentally demanding would using RESCUE-AR be?

Physical Demand: How physically demanding would using RESCUE-AR be?

Temporal Demand: How hurried or rushed would you feel when using RESCUE-AR?

Effort: How hard would you have to work to accomplish your level of performance?

Frustration: How insecure, discouraged, irritated, or stressed would you feel when using RESCUE-AR?

**3. Core Concept and Operational Suitability Questions**

I would trust the information provided by RESCUE-AR during emergency operations.

The combination of multiple sensors (GPS, IMU, SLAM) increases my confidence in the data accuracy.

The visual annotations in Augmented Reality would significantly improve my situational awareness in the field.

A hands-free or low-interaction operation is essential for this type of system in critical environments.
